# Effects of vatinoxan and fentanyl on blood glucose concentrations and diuresis in male Wistar rats sedated with medetomidine and midazolam

**DOI:** 10.1186/s12917-026-05304-2

**Published:** 2026-01-29

**Authors:** Emily Lindh, Anna Meller, Karoliina Alm, Marja Raekallio, Juhana Honkavaara

**Affiliations:** 1 Faculty of Veterinary Medicine, Department of Equine and Small Animal Medicine, University of Helsinki, PO Box 57, Helsinki, FIN-00014 Finland; 2 HiLIFE Institute, Laboratory Animal Center, University of Helsinki, Helsinki, Finland

**Keywords:** Alpha2-agonist, Alpha2-antagonist, Hyperglycemia, Polyuria, Glucosuria, Rats, Vatinoxan

## Abstract

**Background:**

Alpha2-adrenoceptor agonists such as medetomidine induce hyperglycemia, glucosuria, and polyuria. We evaluated the ability of vatinoxan, a peripherally acting alpha2-adrenoceptor antagonist, to mitigate these side effects in male Wistar rats (*N* = 31). To explore its impact on two common sedation protocols in laboratory rats, a randomized, controlled experimental study was conducted comprising four groups: medetomidine (0.25 mg/kg) and midazolam (2.0 mg/kg) (MM; *N* = 8), MM + fentanyl (0.01 mg/kg) (MMF; *N* = 8), MM vatinoxan (MMV; *N* = 7), and MMF + vatinoxan (MMFV; *N* = 8). Blood glucose concentration (BG) was measured repeatedly between 10 and 60 min after treatment administration, and total urine output and body weight change were assessed. Additionally, urine glucose concentration (UG) was measured at 45 and 75 min.

**Results:**

Pooled BG was significantly higher in non-vatinoxan treated rats [19.8 ± 3.6 mmol/L (mean ± SD) with MM + MMF compared with 10.3 ± 1.2 mmol/L with MMV + MMFV] (*P* < 0.001). Similarly, urine output was greater without vatinoxan [median 22.9 (minimum–maximum) 10.6–32.9) vs. 2.8 (0.0–7.4) ml/kg/h] (*P* < 0.001). Vatinoxan also reduced UG [33.3 (0.6–33.3) vs. 1.4 (0.9–3.2) mmol/L] (*P* < 0.01) and body weight loss. Fentanyl did not significantly alter these outcomes. Rats were sufficiently sedated after all treatments.

**Conclusions:**

The findings demonstrate that vatinoxan effectively mitigates hyperglycemia, polyuria and glucosuria induced by medetomidine-based sedation protocols in adult, male Wistar rats.

**Supplementary Information:**

The online version contains supplementary material available at 10.1186/s12917-026-05304-2.

## Background

Alpha2-adrenergic agonists (α2-agonists) such as dexmedetomidine, medetomidine and xylazine are commonly used in laboratory rodent sedation. Benzodiazepines and opioid receptor agonists, such as fentanyl are commonly added to improve sedation and antinociception [1, 2]. A combination of medetomidine, midazolam, and fentanyl offers reliable and reversible sedation in laboratory rats [3]. However, α2-agonists have extensive influence on metabolic and endocrine functions. They reduce insulin release from pancreatic β-cells [4], resulting in hyperglycemia [5]. Additionally, α2-agonists induce polyuria in various species, including rats [6–12], dogs [13, 14], and horses [15]. These agents are thought to increase urine excretion mainly by inhibiting the secretion of antidiuretic hormone (ADH) in the hypothalamus [12, 16] and by blocking ADH’s effects on the distal renal tubules and collecting ducts [9, 17]. Furthermore, α2-agonists increase the concentration of natriuretic peptide in plasma in dogs [18] and rats [19]. α2-agonists also suppress renin production in the kidneys, which increases urine volume via reducing aldosterone-mediated water reabsorption [20, 21]. Urine output may also be increased by α2-agonist-induced glucosuria and consequent osmotic diuresis [14]. In dogs, both hyperglycemia and polyuria can be reversed with αlpha2-adrenoceptor antagonists (α2-antagonists) such as yohimbine and atipamezole [22].

Vatinoxan (also known as MK-467 or L659,066) is a hydrophilic α2-antagonist that does not readily cross the blood-brain barrier [23]. Consequently, its impact on the central action of α2-agonists appears minimal and mainly related to its effects on the disposition of co-administered drugs [24, 25]. In horses receiving detomidine, vatinoxan alleviated α2-agonist-induced hyperglycemia, glucosuria and maintained insulin secretion [26, 27]. Vatinoxan alone decreased urine output in hydrated Wistar rats [28] and preliminary reports suggest that it might migitate hyperglycemia in rats sedated with medetomidine-based protocols [11, 29]. A preformulated mixture containing both medetomidine and vatinoxan is commercially available in the U.S. and European markets and is approved for intramuscular sedation in dogs for short non-painful procedures.

The aim of this study was to investigate the effects of vatinoxan on blood and urine glucose concentration (BG and UG), urine output and body weight loss in male Wistar rats sedated with medetomidine and midazolam, with or without fentanyl. Our hypotheses were that vatinoxan would alleviate the medetomidine-induced hyperglycaemia, decrease UG, and prevent polyuria and body weight reduction, without affecting the medetomidine-induced sedation. We further hypothesized that the addition of fentanyl would not significantly affect these outcomes.

## Methods

This terminal study involved 32 healthy male Wistar rats, aged 10 weeks and weighing between 339 and 410 g. Purpose-bred animals were purchased from Envigo RMS B.V., Netherlands. All procedures were approved by the Project Authorization Board, operating under the Regional State Administrative Agency in Finland (license ESAVI/39801/2023). The license matches the requirements of both the European Union legislation and the updated ARRIVE 2.0 guidelines for reporting in vivo experiments [30].

The rats were housed in pairs within individually ventilated cages (Blue Line 1500U for Rats, Tecniplast, Italy) under specific pathogen-free conditions at the University of Helsinki Laboratory Animal Centre and maintaining a temperature of 21 °C ± 1 °C, humidity at 55% ± 10%, and a 12-hour light/dark cycle with lights on at 6:00 a.m. Animals had unrestricted access to a standard rodent diet (Teklad Rodent Diet 8460, Inotiv, IN, USA) and purified water *ad libitum.*

Animals were randomly assigned to one of four treatment groups (*N* = 8 per group):

(1) medetomidine hydrochloride (HCl) 0.25 mg/kg (Dorbene 1 mg/ml, Laboratorios Syva S.A, Spain) combined with midazolam HCl 2 mg/kg (Midazolam Hameln 5 mg/ml, Hameln Pharma GmbH, Germany) (MM), (2) MM + vatinoxan HCl 5 mg/kg (medetomidine HCl 0.5 mg/ml and vatinoxan HCl 5 mg/ml, Zenalpha 0.5 mg/ml and 10 mg/ml, Apotek Produktion & Laboratorier AB, Sweden) (MMV), (3) MM + fentanyl HCl 0.01 mg/kg (Fentanyl Hameln 0.05 mg/ml, Hameln Pharma GmbH, Germany) (MMF), or (4) MMF combined with vatinoxan HCl 2.5 mg/kg (MMVF).

Dosages were selected based on prior research and aimed to produce reliable sedation throughout the 75-minute experimental period. Drugs were freshly prepared each day before trials, and diluted with saline to a standardized injection volume of 1.1 ml/kg.

The rats were weighed with a dynamic precision scale (Mettler Toledo JE3200, Mettler-Toledo, LLC., OH, USA) before administering the treatments subcutaneously (SC) in the caudal section of the flank. Treatments were delivered in a single syringe and a 25 G 16 mm hypodermic needle (time 0 min), after which the rat was placed into an individual cage until recumbent.

**Fig. 1:**
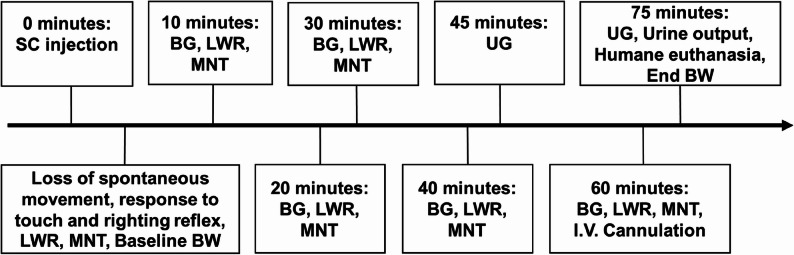
Study timeline. LWR = leg withdrawal reflex, MNT = mechanical nociceptive threshold, BW = body weight, BG = blood glucose concentration, UG = urine glucose concentration

All assessments were always performed by the same investigator, who was kept blinded to the treatments until the end of study. Figure 1 presents the detailed timeline of the measurements. The time to cessation of spontaneous movement was recorded. After that, the time to loss of response to the touch of the rat’s flank was recorded by pushing the flank of the rat’s caudal portion of the body with a finger (assessed every 30 s after cessation of spontaneous movement). The response to touch was assessed as lost when no noticeable response i.e. head or tail movement or return to ambulation was noted. Once the rat became unresponsive to touch, the loss of righting reflex was assessed every 60 s. The righting reflex was classified as absent, when all four of the rat’s paws remained upwards after turning the rat from sternal recumbency to dorsal recumbency. After the righting reflex was lost, antinociception was tested by measuring the response threshold to mechanical pressure stimulation (MNT) on the left metatarsal area (Digital Paw Pressure Randall Selitto Meter, IITC Life Science Inc., CA, USA) [31]. A cut-off value of 400 g for the leg withdrawal threshold was chosen following previous studies that adopted the manufacturer’s recommended maximum force for reliable testing and to avoid tissue damage [31]. If the upper threshold was reached, the stimulus was discontinued immediately. Finally, the presence of the leg withdrawal reflex (LWR) was assessed by pinching a digit on the right hindfoot while simultaneously extending the leg. The test was performed using the blinded investigator’s thumb and forefinger nails. The skin between the second and third digits on the right foot was pinched once with gradually increasing force to avoid causing skin damage. LWR was considered absent if no flexion of the leg muscles were observed after a single pinch. Weighing was then repeated before placing the rat in dorsal recumbency on a pre-weighed absorbent pad.

Body temperatures were maintained with a heating pad (Far Infrared Surgical Warming Pad DCT-25, Kent Scientific Co. CO, USA) and bubblewrap. The rats were breathing room air throughout the study.

Blood samples for BG measurements were obtained from a vein on the hind foot utilizing a and UG concentration was measured (Ascensia Contour Care Blood Glucose Monitoring System, Ascensia Diabetes Care Holdings AG, Switzerland) if a sample could be obtained from spontaneous overflow or by gentle manual expression, utilizing a 0.6 µL test strip. Rats were cannulated into the tail vein after 60 min (24 G 19 mm Vasofix Certo, B.Braun, Melsungen AG, Germany or 26 G 19 mm Terumo Versatus-W, Terumo, Japan), and at 75 min rats were euthanized with 50 mg/kg pentobarbital (Euthoxin 400 mg/ml, Chanelle Pharmaceuticals Manufacturing Ltd., Ireland). All animals’ bladders were expressed to collect any remaining urine onto the absorbent pad, ensuring comparable urine output assessment across animals. Rats were then weighed post-mortem to determine body weight change from baseline. Following this, death was confirmed by cervical dislocation. Finally, the absorbent pad from under the rat was weighed to assess urine output [32]. Urine output was calculated as the total volume of urine collected from each animal over the 75-minute sampling period (spontaenously voided and manually expressed). The volume was normalized to body weight and expressed as mL/kg/hour using the following formula:

$$\begin{aligned}&\text{Urine output (mL/kg/h)}=\\&\frac{\text{Total urine volume (mL)}}{\text{Body weight (kg)}\times\:\text{Collection time (h)}}\end{aligned}$$

With a power of 80% and an alpha-level set at 0.05, seven animals were required to detect a 10 mmol/L (SD 5 mmol/L) difference in BG between treatments. The individual animal was considered the experimental unit for all analyses. Statistical Package for Social Sciences (SPSS version 29 0.2.0 IBM, NY, USA) was used for all statistical analysis. We expected seven animals to further suffice in detecting a 10 ± 5 ml/kg/h difference in urine output. For UG, urine output and body weight reduction the Kruskal-Wallis test was used for the effect of treatment, between MM and MMV, MMF and MMFV, and between MM and MMF and MMV and MMFV, followed by a post-hoc Dunn’s test for multiple pairwise comparisons where applicable. For the analysis of BG, a one-way analysis of variance was applied for the effect of treatment, followed by independent samples t-tests for pairwise comparisons between the vatinoxan and non-vatinoxan treatment, as our a priori hypothesis focused specifically on the effect of vatinoxan. Multiple comparisons were corrected with the Bonferroni *post hoc* adjustment. Results were considered statistically significant when *P* < 0.05.

## Results

**Fig. 2:**
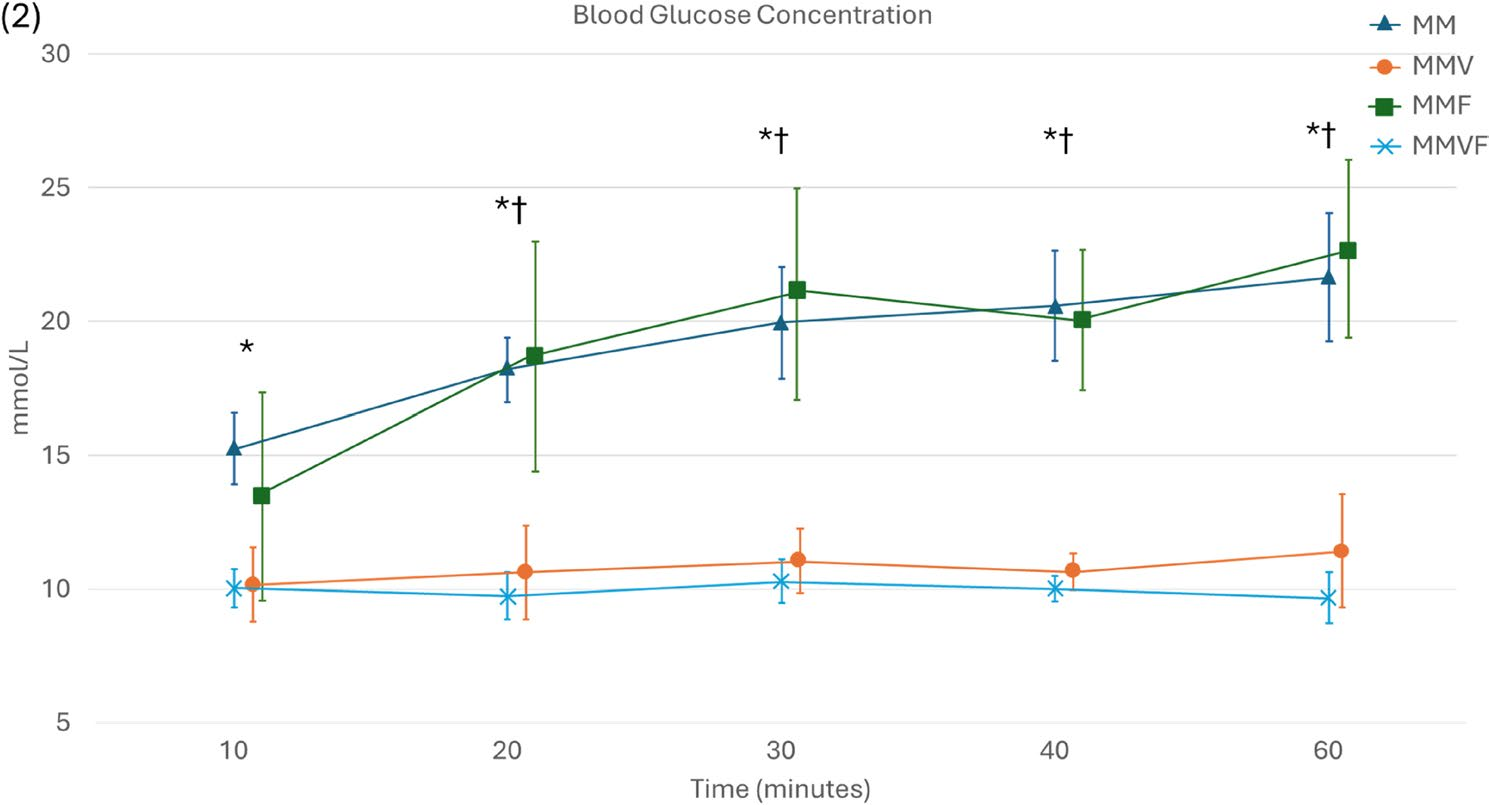
Blood glucose concentrations in Wistar rats administered with medetomidine 0.25 mg/kg and midazolam 2 mg/kg (MM) *N* = 8, MM + vatinoxan 2.5 mg/kg (MMV) *N* = 7, MM + fentanyl 0.01 mg/kg (MMF) *N* = 8 or MMF + vatinoxan 2.5 mg/kg (MMVF) *N* = 8. The data is presented as mean ± SD. * Significant difference at P < 0.001 between MM and MMV. † Significant difference at P < 0.001 between MMF and MMFV

**Fig. 3:**
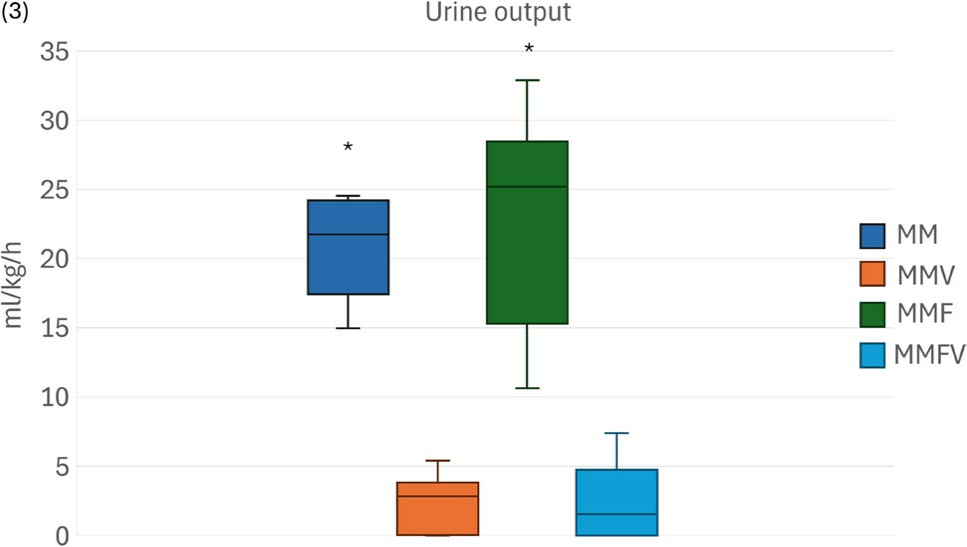
Urine output in Wistar rats administered with medetomidine 0.25 mg/kg and midazolam 2 mg/kg (MM) *N* = 8, MM + vatinoxan 2.5 mg/kg (MMV) *N* = 7, MM + fentanyl 0.01 mg/kg (MMF) *N* = 8 or MMF + vatinoxan 2.5 mg/kg (MMVF) *N* = 8. The data are presented as median and interquartile range (boxes) and minimum–maximum (whiskers). * Significant difference between MM vs. MMV, P = 0.005 and MMF vs. MMFV, P = 0.001)

Thirty-one rats completed the study, with one rat in the MMV group excluded due to a dosing error. In general, BGs were higher in animals that did not receive vatinoxan (Fig. 2). A similar pattern was observed in urine output, with greater volumes in the groups without vatinoxan (Fig. 3).

The number of urine samples (N) analyzed for glucose concentrations varied, as urine could not be collected from all rats in both time points, therefore data from T45 are not presented. However, the UG at 75 min was significantly greater without vatinoxan (Table 1.). Additionally, the loss of body weight was greater in both non-vatinoxan groups. No differences in BG, UG, or BW change were observed between MM and MMF, or MMV and MMVF.

**Table 1:** Urine glucose concentration at 75 min post-injection, urine output and body weight decrease from the onset of sedation until 75 min post-injection in Wistar rats administered with Medetomidine 0.25 mg/kg and Midazolam 2 mg/kg (MM) *N* = 8, MM + vatinoxan 2.5 mg/kg (MMV) *N* = 7, MM + fentanyl 0.01 mg/kg (MMF) *N* = 8 or MMF + vatinoxan 2.5 mg/kg (MMVF) *N* = 8. The number of urine samples (N) analyzed for glucose concentrations varied, as urine could not be collected from all rats

Treatment	Urine glucose concentration (mmol/l) T75	*N*	*p*-value	Decrease of body weight (%)	*p*-value	*N*
MM	33.3 (15.0–33.3)	7		2.4 (1.7–3.4)		8
MMV	1.3 (0.9–1.7)	5	0.012	0.2 (0–1.0)	0.004	7
MMF	33.3 (0.6–33.3)	8		2.7 (1.0–3.6)		8
MMVF	1.4 (0.9–3.2)	7	0.044	0.2 (0–0.7)	0.002	8

The data is presented as median (minimum–maximum). The p-values indicate the difference with the respective treatment without vatinoxan

All rats lost their spontaneous movements, response to touch and righting reflex after each treatment and stayed dorsally recumbent until the end of the monitoring period (75 min). The median times for onset of sedation are presented in Supplementary Tables 1 and median MNTs in Supplementary Fig. 1. LWR was present in all rats throughout the study.

## Discussion

The key finding of this study was that the addition of vatinoxan effectively prevented the hyperglycemia, polyuria and glucosuria observed after both medetomidine-based sedation protocols. Moreover, reduction in body weight was prevented with the addition of vatinoxan. These outcomes appeared independent of the presence of fentanyl. Throughout the 75-minute study period, all rats were adequately sedated after each treatment, as evidenced by consistent loss of righting reflex.

In non-vatinoxan treated rats, medetomidine produced notable hyperglycemia consistent with the well-documented effects of α₂-agonists across several mammalian species [11, 26, 29, 33–37]. This response is primarily mediated by inhibition of pancreatic insulin secretion through stimulation of peripheral α₂-adrenoceptors, reducing cellular uptake of glucose from blood as also demonstrated in rodents [38–40]. By preventing this adverse outcome, vatinoxan may enable the use of α₂-agonists in e.g. research models and animals that do not tolerate hypoinsulinemia, hyperglycemia and glucosuria. It is worth noting, however, that the present data was obtained over a relatively short time period, offering only a brief insight into the BG dynamics following α2-agonist exposure in laboratory rats. In equine studies, administration of detomidine, another α₂-agonist, was associated not only with prolonged hyperglycemia but also with a subsequent surge in insulin secretion, rebound hyperinsulinemia [26]. As such, while transient hyperglycemia was evident, whether rats experience a similar pattern with glucose and insulin later on remains unclear.

The urine output of non-vatinoxan treated rats was significantly greater compared with those receiving vatinoxan. This finding is consistent with medetomidine-induced polyuria reported previously [11, 14, 15]. Diuresis following α2-agonists can last for 2 h in horses [15] and up to 4 h in dogs [14]. In the absence of vatinoxan, the hourly rate of urine output was nearly 1/3 of the rat’s daily fluid requirements, similar to a previous report [11].

There are several mechanisms underlying α₂-agonist-induced polyuria. The principal mechanism is inhibition of the ADH secretion in the hypothalamus [41] accompanied by modulation of the renal response to ADH at the level of the collecting ducts [42]. In addition, renal glucose overflow may contribute once the tubular reabsorption threshold is exceeded, resulting in osmotic diuresis [43]. However, increased diuresis without glucosuria has been observed in dogs and horses administered medetomidine or detomidine, respectively [44, 45]. In this study, vatinoxan prevented the substantially high UGs. This finding confirms that the renal tubular threshold for glucose was exceeded [46, 47].

In this study, vatinoxan prevented the significant reduction in body weight during the relatively short observational period. The rate of weight loss cannot be explained by the moderate reduction (< 3%) in body weight that would be expected during any post-operative period due to changes in food and water intake [48]. In view of this, fluid replacement therapy is strongly advocated for α2-agonist treated rats even after short periods of sedation. Overall, vatinoxan may improve not only the physiological wellbeing but also for the reliability of experimental outcomes especially in survival studies [49, 50].

The major limitation of this study was that the sample size was calculated for BG and therefore potentially underpowered to detect significant differences in outcomes such as sedation and antinociceptive variables. Vatinoxan affects the disposition of co-administered drugs, which may have potentially increased the variation within the treatment groups [24, 25]. Furthermore, we did not confirm directly how vatinoxan produced its effects, by measuring e.g. insulin or ADH concentrations in plasma. In addition, the limited accuracy and repeatability of MNT, likely due to inconsistent probe placement over the metatarsal area and/or variable pressure application, may have hindered detection of significant treatment effects, including those of fentanyl. Fentanyl, as an opioid receptor agonist has previously been shown to potentiate antinociceptive effects of α2-agonists [51]. However, the level of clinical sedation appeared sufficient throughout our 75-minute study period. Moreover, another key limitation of this study was the use of single-sex subjects. Given the growing emphasis on sex as a biological variable in research design [52], future studies should include female subjects to determine whether the observed effects of vatinoxan are consistent across sexes. Additionally, further investigation is warranted to evaluate the long-term glycemic impacts of vatinoxan administration, such as later fluctuations of BG and insulin, as this study focused only on short-term outcomes.

Vatinoxan effectively mitigated medetomidine-induced hyperglycemia and glucosuria in rats sedated with subcutaneous medetomidine and midazolam. Moreover, vatinoxan prevented medetomidine-induced polyuria and the reduction in body weight. All rats were sufficiently sedated after all treatments. The addition of fentanyl did not alter these outcomes. Vatinoxan may improve medetomidine-based sedation protocols in rats.

## Supplementary Information

Supplementary Material 1.

## Data Availability

The datasets used and/or analysed during the current study are available from the corresponding author on reasonable request.
