# Supplementary material for: Effects of vatinoxan and fentanyl on blood glucose concentrations and diuresis in male Wistar rats sedated with medetomidine and midazolam
Source: BMC Vet Res. 2026 Jan 29;22:119. doi: 10.1186/s12917-026-05304-2 (PMC12924401; doi:10.1186/s12917-026-05304-2)

**Supplementary Table 1.** Onset of sedation in Wistar rats administered with medetomidine 0.25 mg/kg and midazolam 2 mg/kg (MM) N=8, MM + vatinoxan 2.5 mg/kg (MMV) N=7, MM + fentanyl 0.01mg/kg (MMF) N=8 or MMF + vatinoxan 2.5 mg/kg (MMVF) N=8.

| Treatment | Loss of spontaneous movement (seconds) | Loss of response to touch (seconds) | Loss of righting reflex (seconds) |
| --- | --- | --- | --- |
| MM N=8 | 340  (90–550) | 443  (120–750) | 540  (130–1490) |
| MMV N=7 | 210  (135–580) | 300  (165–610) | 330  (225–1080) |
| MMF N=8 | 334  (137–520) | 419  (167–570) | 623  (187–970) |
| MMVF N=8 | 155  (85–490) | 245  (150–500) | 310  (164–686) |

Data is presented as median (minimum–maximum).

**Supplementary Figure 1.** Mechanical nociceptive threshold (MNT) at the time of loss of the righting reflex (LoRR) and until 60 minutes in Wistar rats administered with medetomidine 0.25 mg/kg and midazolam 2 mg/kg (MM) N=8, MM + vatinoxan 2.5 mg/kg (MMV) N=7, MM + fentanyl 0.01mg/kg (MMF) N=8 or MMF + vatinoxan 2.5 mg/kg (MMVF) N=8.

The data are presented as median and interquartile range (boxes) and minimum–maximum (whiskers). No significant differences were detected between treatments.


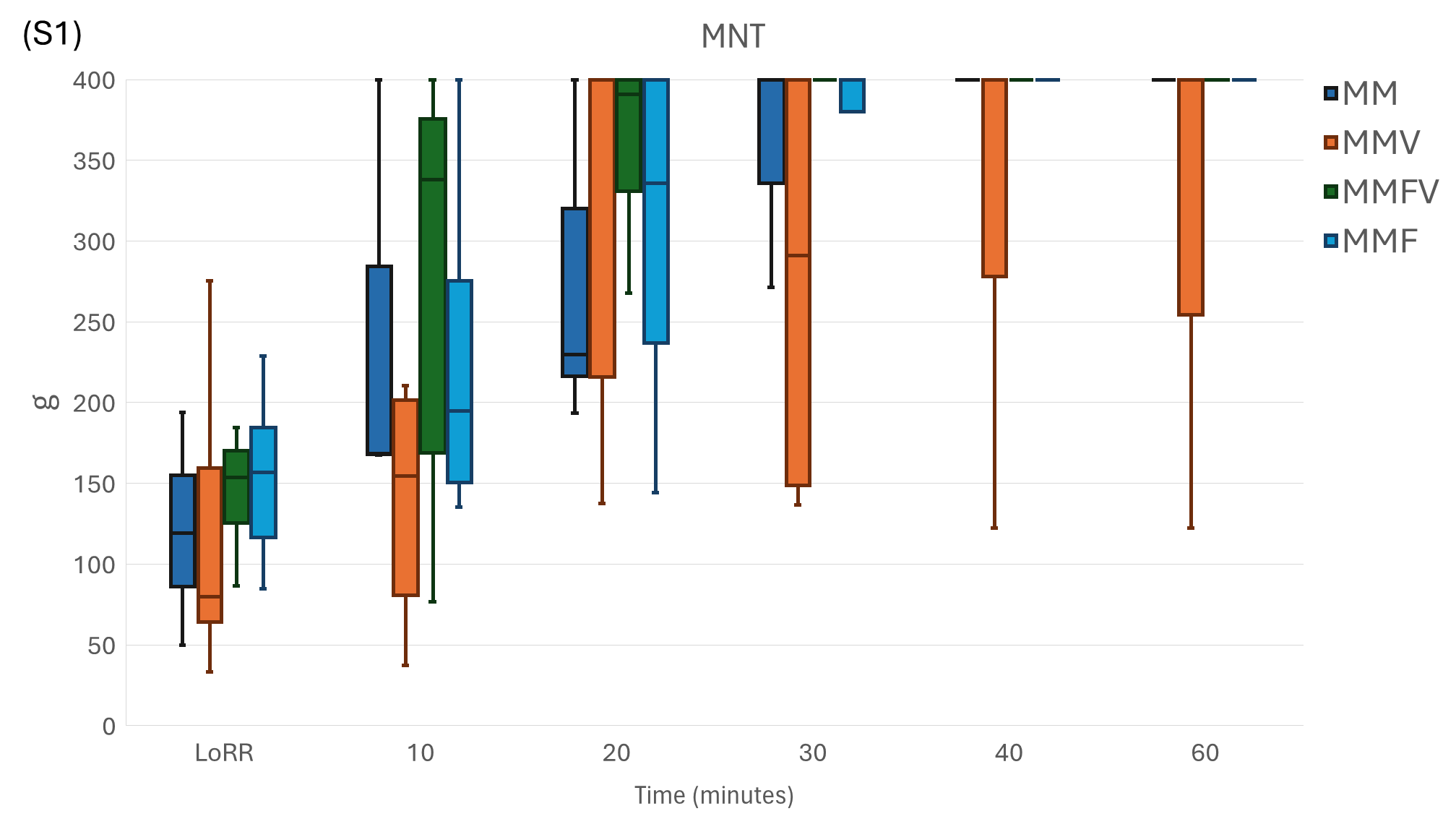

Supplement: Supplementary file 1 — Supplementary Material 1. [file 12917_2026_5304_MOESM1_ESM.docx]
